# Neuroanatomical Abnormalities in Violent Individuals with and without a Diagnosis of Schizophrenia

**DOI:** 10.1371/journal.pone.0168100

**Published:** 2016-12-28

**Authors:** Victor A. Del Bene, John J. Foxe, Lars A. Ross, Menahem I. Krakowski, Pal Czobor, Pierfilippo De Sanctis

**Affiliations:** 1 The Sheryl and Daniel R. Tishman Cognitive Neurophysiology Laboratory Children’s Evaluation and Rehabilitation Center (CERC) Departments of Pediatrics and Neuroscience Albert Einstein College of Medicine Van Etten, New York, United States of America; 2 Ferkauf Graduate School of Psychology Albert Einstein College of Medicine Bronx, New York, United States of America; 3 The Nathan S. Kline Institute for Psychiatric Research Orangeburg, NY, United States of America; 4 The Ernest J. Del Monte Institute for Neuromedicine Department of Neurobiology and Anatomy University of Rochester Medical Center Rochester, New York, United States of America; 5 New York University Langone Medical Center Department of Psychiatry New York, New York, United States of America; 6 Departments of Psychiatry and Psychotherapy Semmelweis University ÜllőiWay 26, Budapest, Hungary; 7 Center for Psychiatric Neuroscience The Feinstein Institute for Medical Research Manhasset, NY, United States of America; 8 Department of Psychiatry Hofstra Northwell School of Medicine Zucker Hillside Hospital Glen Oaks, NY, United States of America; Universidad de Jaen, SPAIN

## Abstract

Several structural brain abnormalities have been associated with aggression in patients with schizophrenia. However, little is known about shared and distinct abnormalities underlying aggression in these subjects and non-psychotic violent individuals. We applied a region-of-interest volumetric analysis of the amygdala, hippocampus, and thalamus bilaterally, as well as whole brain and ventricular volumes to investigate violent (n = 37) and non-violent chronic patients (n = 26) with schizophrenia, non-psychotic violent (n = 24) as well as healthy control subjects (n = 24). Shared and distinct volumetric abnormalities were probed by analysis of variance with the factors violence (non-violent *versus* violent) and diagnosis (non-psychotic *versus* psychotic), adjusted for substance abuse, age, academic achievement and negative psychotic symptoms. Patients showed elevated vCSF volume, smaller left hippocampus and smaller left thalamus volumes. This was particularly the case for non-violent individuals diagnosed with schizophrenia. Furthermore, patients had reduction in right thalamus size. With regard to left amygdala, we found an interaction between violence and diagnosis. More specifically, we report a double dissociation with smaller amygdala size linked to violence in non-psychotic individuals, while for psychotic patients smaller size was linked to non-violence. Importantly, the double dissociation appeared to be mostly driven by substance abuse. Overall, we found widespread morphometric abnormalities in subcortical regions in schizophrenia. No evidence for shared volumetric abnormalities in individuals with a history of violence was found. Finally, left amygdala abnormalities in non-psychotic violent individuals were largely accounted for by substance abuse. This might be an indication that the association between amygdala reduction and violence is mediated by substance abuse. Our results indicate the importance of structural abnormalities in aggressive individuals.

## Introduction

There is a growing interest to further our understanding of violence and aggression in patients with severe mental illness. Violent behavior is a public health concern, and there is evidence of increased rates of violence in patients with schizophrenia [1–3]. Although violence is determined by multiple factors, emerging evidence suggests that emotional regulation, the ability to modulate one’s state or behavior in a given situation plays an important role in aggressive behavior [4–6]. Here, we investigate brain structures associated with emotional regulation comparing schizophrenia patients with (VS) and without a history of violence (NVS) as well as non-psychotic violent individuals (NPV) to delineate neuroanatomical abnormalities linked to psychosis and aggression.

Converging evidence from focal brain lesion studies [7], functional imaging [5, 8] and neurotransmitter functioning [9–11] in aggressive individuals implicates the amygdala in the etiology of violence, with the amygdala considered a key structure in several neurobiological models of violence [12–14]. For example, the Siever model [14] proposes that violence and aggression occur when amygdala-mediated affective responses are not inhibited, and this lack of inhibition prevents the impulsive drive from being constrained. Deficits in inhibition, along with perceived urgency and difficulties in self-regulation associated with prefrontal cortex dysfunction increase the risk of violence [15–18].

Studies of patients with schizophrenia have shown aberrant brain structures and functions in limbic and frontal regions. The hippocampus and whole brain volume are consistently found to be reduced in volume in patients with schizophrenia [19–23], particularly so in patients with a history of violence [24–27]. Applying resting-state functional magnetic resonance imaging (fMRI), Hoptman and colleagues [28] showed reduced functional connectivity (FC) between the amygdala and ventral prefrontal regions in patients with schizophrenia, with lower connectivity associated with higher levels of self-rated aggression. Recently, we used event-related brain potentials to show a delay of approximate 150 millisecond in sensory-perceptual responses to emotionally salient stimuli in schizophrenia patients with a history of violence relative to their non-violent peers [29]. Given this deficit is related to the processing of negatively valenced stimuli, aberrant amygdala functioning must be considered. Hence, there is consistent evidence of aberrant brain functions linked to emotional regulation and its role in aggression.

Interestingly, structural imaging studies of the amygdala in violent patients with schizophrenia have yielded mixed results. One early study found temporal lobe volume reduction in violent patients [30] in contrast to more recent work [24–26, 31]. Barkataki and colleagues [24] found that non-violent patients showed amygdala reductions relative to healthy controls and violent patients, but differences between patient groups were lost after controlling for positive symptoms. Similar results were reported in two more investigations with amygdala reductions seen in non-violent patients [25, 26]. One of the studies also reported a weak trend towards a reduction in violent patients compared to healthy controls [25]. All the above studies unfortunately are characterized by small sample sizes (ranging from 10 to 13 for violent patients), which might contribute to these inconsistent findings. Considering the rather robust functional evidence implicating amygdala in aggression, further structural studies with larger sample sizes are clearly warranted.

The relationship between amygdala reduction and violence appears to be somewhat more consistent in non-clinical samples, which raises the important question about differential neuronal contributions to violence. Using the symptom check list to confirm the absence of psychiatric disorders (SCL-90-R)[32], Matthies and colleagues classified individuals as aggressive based on their Lifetime History of Aggression score (LHA > 5)[33]. They reported smaller bilateral amygdala volumes in aggressive individuals. Lending support to the specificity of their findings, amygdala size correlated with LHA scores, but not with other clinical measures looking at general psychopathology, anxiety, and depression. Similar results were reported by Bobes Leon et al [34] applying the Reactive and Proactive Aggression Questionnaire [35] to measure aggressiveness in adult men. Amygdala size was found to be smaller in the violent group and negatively correlated with trait scores of reactive aggression. Notably, total score on the psychopathy checklist for the violent group at 10.6 was far below the commonly used cut-off score of 25 for psychopathy [36]. In a recent longitudinal study, amygdala volumes measured at 26 years of age were associated with psychopathic features and levels of aggression measured in youth. Yet, group volumetric differences between adult individuals with a history of chronic, transient, and no serious violence were not significant [37].

Investigations on the issue of violence in schizophrenia have produced promising results, but studies with small sample size and inclusion of comorbid disorders [24–26, 31] make interpretation of volumetric changes less clear. Including a non-psychotic, violent sample without a history of any major Axis I disorder, or any current substance abuse problems will help distinguish neuroanatomical abnormalities associated with aggression and psychosis.

## Methods

### Participants

Region of interest (ROI) analysis for non-violent patients with schizophrenia (NVS; *n* = 26), violent patients with schizophrenia (VS; *n* = 37), non-psychotic, violent participants (NPV; *n* = 24), and healthy control participants (HC; *n* = 24) were carried out (for list of abbreviations see Table 1). All participants, as reported in our previous studies using the same participants [29, 38], had no significant co-morbid medical, psychiatric, or neurological illnesses. Participants with any history of drug or alcohol dependence were excluded, along with any recent drug or alcohol abuse occurring 6 months or less to their participation in the study. The structured clinical interview for DSM-IV-TR disorders (SCID-IV DSM-IV-TR)[39, 40] was used for diagnostic purposes for all four groups, including the confirmation that our HC and NPV participants did not meet criteria for any Axis 1 psychiatric disorders. The patient population was recruited from the Rockland Psychiatric Center (Rockland, NY). In particular, patients were recruited from the Nathan S. Kline Institute (NKI) inpatient facilities, as well as outpatient centers in New York State. Non-patient participants were recruited from the volunteer recruitment pool at NKI. Informed written consent was obtained from all participants. NKI IRB approved consent forms were used for this process. The experimental protocol was approved by the institutional review board at NKI and is compliant with the tenets set forth in the Declaration of Helsinki.

**Table 1:** Abbreviations.

Healthy Controls	HC
Nonviolent patients with schizophrenia	NVS
Violent patients with schizophrenia	VS
Non-psychotic violent participants	NPV
Whole Brain Volume	WBV
Ventricular Cerebrospinal Fluid	vCSF
Positive and Negative Syndrome Scale	PANSS
PANSS General Score	panGS
PANSS Total Score	panTS
PANSS Positive Score	panPS
PANSS Negative Score	panNS
Substance Abuse Disorder	SUD
Wide Range Achievement Test	WRAT-4
Life History of Aggression	LHA
Barratt Impulsiveness Scale-11	BIS-11

### Clinical Assessment

The Life History of Aggression (LHA) [41] form was completed for all participants to help determine grouping (e.g. violent or non-violent). Participants report during interview, available official criminal records ("Record of Arrest and Prosecution"), staff interviews, and chart review was used to complete the LHA. The LHA scale has strong inter-rater and test-retest reliability, good internal consistency, and is considered a valid measure of overt aggression [41]. Scores ≥ 20 on the LHA, plus a confirmed episode of physical assault in the past year, in conjunction with a score ≥ 3 on the "Physical Aggression Against People" (PA-people) subscale, met criteria for inclusion in the two violent groups (e.g. VS and NPV). To be included in the non-violent group, there could be no evidence of a physical assault within the past year, or any lifetime episodes of severe physical aggression, accompanied with a score ≤ 15 on the LHA, and ≤ 2 on the PA-people. This study used the same inclusion and exclusion criteria found in our previous studies [29, 38].

The Buss-Perry Aggression Questionnaire [42], is a 29-item measure, comprised of four dimensions (physical aggression, verbal aggression, hostility, and anger). There is moderate to high internal consistency, with stable test-retest scores over 7 months, and it has good convergent validity, although there is a moderate negative relationship with social desirability [43].

The Barratt Impulsiveness Scale (BIS-11) [44] is a widely used measure of impulsive personality traits that is multi-dimensional with strong internal consistency, test-retest reliability, and convergent validity [45].

The Wide Range Achievement Test–Fourth Edition, Reading Subtest (WRAT-4) was used to estimate academic achievement skills and IQ [46]. Age-adjusted T-scores were used for all analyses.

The Positive and Negative Syndrome Scale for Schizophrenics (PANSS) [47] was used to help characterize the schizophrenia patient population. The PANSS has been found to have good psychometric properties with no inter-correlation between the negative and positive scales suggesting separate dimensions of psychopathology [47, 48]. Only patients with schizophrenia were administered the PANSS.

### Neuroimaging Acquisition and Data Processing

T1-weighted magnetic resonance (MR) images were acquired with a 1.5T Siemens Vision scanner (Erlangen, Germany) at the NKI Center for Advanced Brain Imaging (Orangeburg, NY), which was equipped with a 30.5-cm i.d. three-axis local gradient coil and an end-capped quadrature birdcage radio-frequency coil. All participants were scanned with the following high-resolution T1-weighted magnetization prepared rapidly acquired gradient echo (MPRAGE) parameters: TR = 11.6 ms, TE = 4.9 ms, flip angle 8°, FOV = 300 mm, matrix = 256x256, slice thickness = 1.2 mm isotropic voxels, and 172 sagittal slices.

Individual volumes were concatenated using AFNI (to3d function; NIMH Bethesda, MD, USA, http://afni.nimh.nih.gov/afni/) to create a single 3D MRI image per participant, prior to using FSL (FMRIB—Oxford; http://fsl.fmrib.ox.ac.uk/fsl/fslwiki/) for segmentation. An automatic approach to the segmentation of subcortical structures, called FIRST was used [49, 50] (http://fsl.fmrib.ox.ac.uk/fsl/fslwiki/FIRST). A benefit of using an automated approach is that manual segmentation is time consuming, since it requires the accurate demarcation of structures and associated anatomical landmarks. Automated packages, such as FIRST, can reliably segment volumes in a fraction of the time, and does not require expert neuroanatomists to manually draw masks. FIRST has the capabilities to automatically delineate and segment sub-cortical structures using a Bayesian based model that creates surface meshes on sub-cortical structures. A large training data-set of 336 T1-weighted MR images from the Center for Morphometric Analysis (CMA; Massachusetts General Hospital, Boston, USA) was used to manually segment 15 structures [50]. Among those structures were the left and right amygdala, hippocampus, and thalamus. As part of the subcortical segmentation to MNI space, FIRST applies a 12 degrees of freedom affine registration of the T1 image to the FSL FIRST training library, followed by a second 12 degree of freedom registration using an MNI subcortical mask to ensure voxels outside of the regions of interest are not incorrectly included in the volumes.

In addition to utilizing FIRST, the cross-sectional version of SIENA ("Structural Image Evaluation using Normalization of Atrophy") called SIENAX was used to estimate whole brain tissue volume from a single T1-weighted image. The process segments brain tissue from non-brain tissue [51, 52] before the brain is affine-registered to MNI152 space [53, 54]. Both, the normalized (e.g. MNI 152) and unnormalized (native) values are provided as outputs. This approach also extracts the grey and white matter, peripheral grey matter, and ventricular cerebrospinal fluid (vCSF) volumes using a partial volume estimation approach [55]. Since FIRST uses linear and non-linear transformations to normalize brain tissue prior to segmentation, we used the normalized values for WBV and vCSF for our analyses. SIENAX has been validated to differentiate clinical groups from a single time-point, with a 0.5–1% brain volume accuracy for SIENAX measures, compared with longitudinal SIENA brain volume change error of 0.15% [56]. It has been found that the estimation of the normalized brain volume accuracy of SIENAX is closely correlated with the SIENA measure [57]. vCSF volume was used as a proxy to measure ventricular size in our current study.

Statistical analyses were carried out using SPSS (IBM SPSS Statistics Version 20). Statistical outliers were excluded from analysis if their ROI volume was 2.5 or more standard deviations from the mean. Prior to excluding any outliers, T1-weighted MRI and the segmentation output quality were first verified. If the segmentation did not occur correctly, it was re-processed to ensure that an error did not occur during the segmentation. In this study, there were no segmentation errors. Due to motion artifact on T1-weighted MR images and significant statistical outliers we excluded six participants in total from the analysis (2 HCs, 1 VS, 1 NVS, and 2 NPV). To determine shared and distinct volumetric abnormalities, we applied analysis of variance (ANOVA) to test main and interactive effects between factors violence (non-violent *versus* violent) and diagnosis (non-psychotic *versus* psychotic). ANOVA was adjusted for substance abuse, age, academic achievement and negative psychotic symptoms. Bonferroni was used to correct for multiple post hoc pairwise comparisons.

## Results

### Demographic and Clinical Variables

Demographic statistics are reported in Table 2. No difference in gender was observed between the groups, with the sample being largely comprised of men. No difference was also seen in medication dose, as measured using chlorpromazine equivalency values. Chlorpromazine equivalents were calculated following the guidelines published in [58]. The percentage of atypical and typical antipsychotic medication was 70% and 30%, respectively. There was a significant difference in age among groups (*F*_3,107_ = 2.94, *p* = .036), with NVS, older compared to HC, VS, and NPV. Groups also differed in years of education (*F*_3, 109_ = 10.7, *p*< .001), with HC having spent more years at school than VS, NPV, and NVS. Age at first hospitalization did not differ between the VS and NVS (*t*_*57*_ = 0.799, *p* = .428), although the duration of illness was longer for the NVS group than the VS group (*t*_*56*_ = 2.30, *p* = .025). Differences were also observed for WRAT-4 performances across groups (*F*_3,106_ = 3.82, *p* = .012), with HC participants obtaining higher achievement scores than the other three groups. Age and WRAT-4 were used as covariates in all subsequent ROI analysis, along with substance abuse disorder.

**Table 2:** Demographic and clinical data.

	HC	NVS	VS	NPV	f/chi/t value	*P*-value
*N*	24	26	37	24	-	-
Gender: male/female	19/5	20/6	31/7	23/1	10.03	.124
Age (s.e.m.)	30.04 (2.1)	43.08 (1.9)	35.3 (1.6)	38.8 (2.1)	2.94	.036*
Age of first hospitalization	--	23.94 (6.62)	22.09 (9.37)	--	.799	.428
Illness Duration	--	19.79 (1.89)	13.79 (1.73)	--	2.30	.025*
Education in years	14.6 (0.4)	13.2 (0.4)	11.7 (0.3)	12.4(0.4)	10.70	**.000** ***
Medication dose (CPZ equivalents)	--	1343.7 (687.4)	1230.6 (637.8)	--	0.65	.52
Substance Abuse	2/24	5/26	18/37	14/24	20.011	.000***
WRAT-4	50.3 (1.8)	42.1 (2.1)	45.1(1.2)	43.2 (2.1)	3.82	.012*
LHA Total	13.83 (1.31)	10.28 (.97)	25.61 (.98)	33.13 (1.18)	64.23	.000***
Barratt Impulsivity	55.13 (1.79)	61.27 (2.49)	62.31 (1.85)	65.92 (2.14)	4.12	.008*
Buss-Perry Total	56.46 (2.71)	63.89 (4.23)	77.12 (3.00)	85.17 (5.58)	10.05	.000***
Buss-Perry Physical	15.38 (1.02)	15.64 (1.39)	22.25 (1.19)	26.52 (1.90)	14.02	.000***
Buss-Perry Verbal	13.13 (.86)	11.88 (.94)	14.12 (.83)	16.33 (1.03)	3.89	.011*
Buss-Perry Anger	11.71 (.60)	14.35 (1.07)	18.21 (1.08)	21.54 (1.88)	11.35	.000***
Buss-Perry Hostility	16.13 (1.14)	20.96 (1.66)	22.03 (.94)	21.33 (1.56)	3.99	.01*
panTT	--	78.5 (14.2)	79.1 (12.9)	--	-0.17	.866
panGS	-	38.7 (1.5)	40.5 (1.2)	-	.89	.349
panPS	--	18.7 (5.4)	20.68 (6.4)	--	-1.28	.21
panNG	--	21.1 (5.3)	17.89 (5.4)	--	2.27	**.027** *

ANOVA or t-test for continuous and chi-square test for categorical variables.

* *P* ≤ 0.05

** *P* ≤ 0.001

*** *P* ≤.0001.

S.E.M. = standard error of mean; S.E.M. are the values in parenthesis; CPZ = chlorpromazine; panTT = PANSS Total Score; panGS = PANSS General Score; panPS = PANSS Positive Score; panNG = PANSS Negative Score; LHA = Life History of Aggression

Additionally, we saw group differences on the Buss-Perry Questionnaire Total Score (BPAQ; *F*_3,107_ = 10.05, *p*< .001). BPAQ total scores were higher in VS and NPV relative to HC and NVS. For the physical aggression subscale of the BPAQ, NPV participants scored higher than VS, and both violent groups reported more physical aggression than NVS and HC. For the verbal aggression subscale of the BPAQ, NPV participants scored higher than HC and NVS. Differences between VS and the other three groups did not reach significance. Furthermore, VS and NPV participants scored higher on anger than NVS and HC. Finally, on the hostility subscale, VS, NPV, and NVS participants scored higher than HC. There were no differences between NPV, VS, and NVS groups.

Life history of aggression scores also differed between the four groups (*F*_3, 108_ = 67.49, *p*< .001). The NPV group was more violent than the VS group, with both of these groups scoring higher on the LHA than both the NVS and HC groups. HC participants had elevated LHA scores relative to the NVS group.

There was a main effect of group (*F*_3, 108_ = 3.28, *p* = .024) for Barrett's Impulsivity Index. Follow-up pair-wise comparisons revealed increased impulsivity in VS and NPV groups relative to HC participants. The NVS group did not differ from the HC group, or the two violent groups.

NVS and VS did not differ in PANSS total scores, general scores, or positive symptoms. However, NVS scored significantly higher on negative symptoms (*t*_59_ = 2.27, *p* = 0.02). For this reason, a secondary analysis looking at the effect of negative symptoms as a contributing factor to ROI volume was conducted.

### Region of Interest Volumetric Analysis

**Fig 1:**
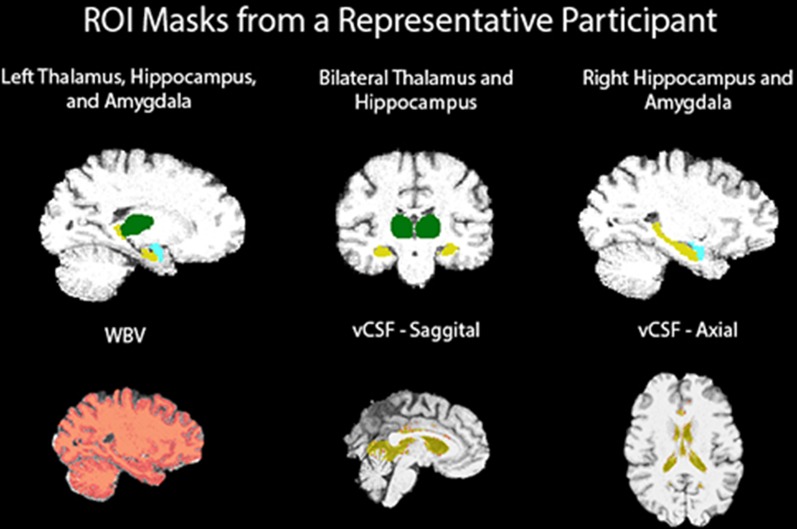
T1-weighted MRI ROI FIRST masks.

Fig 1 illustrates brain regions investigated in our study mapped onto a participant’s brain.

Table 3 lists mean volumes of investigated brain regions for each of the four groups.

**Table 3:** Adjusted means and standard deviations of the means for ROI.

ROI	HC	NVS	VS	NPV
WBV	1610692.14 (118229.65)	1548997.75 (91300.21)	1581467.20 (115945.47)	1562300.89 (79828.58)
Left Amygdala	1290.66 (140.28)	1175.94 (157.60)	1178.25 (241.31)	1218.96 (217.27)
Right Amygdala	1262.81 (139.73)	1128.31 (205.87)	1207.84 (225.95)	1146.76 (304.54)
Left Hippocampus	3669.73 (352.87)	3279.93 (429.43)	3377.83 (426.97)	3328.55 (482.18)
Right Hippocampus	3803.58 (352.95)	3475.02 (321.14)	3557.10 (432.39)	3536.09 (438.81)
Left Thalamus	8345.42 (543.65)	7623.46 (408.86)	8033.83 (640.98)	8034.02 (689.26)
Right Thalamus	8127.71 (618.99)	7559.44 (652.40)	7757.86 (749.18)	7897.09 (653.89)
vCSF	10.59 (0.048)	10.63 (0.05)	10.94 (0.052)	10.75 (0.038)

Adjusted means (S.D.) reported in mm^3^. All ROIs were adjusted for age, substance abuse, and WRAT-4 T-scores.

#### Whole Brain Volume

The ANOVA for WBV revealed no significant main and interactive effects.

**Fig 2:**
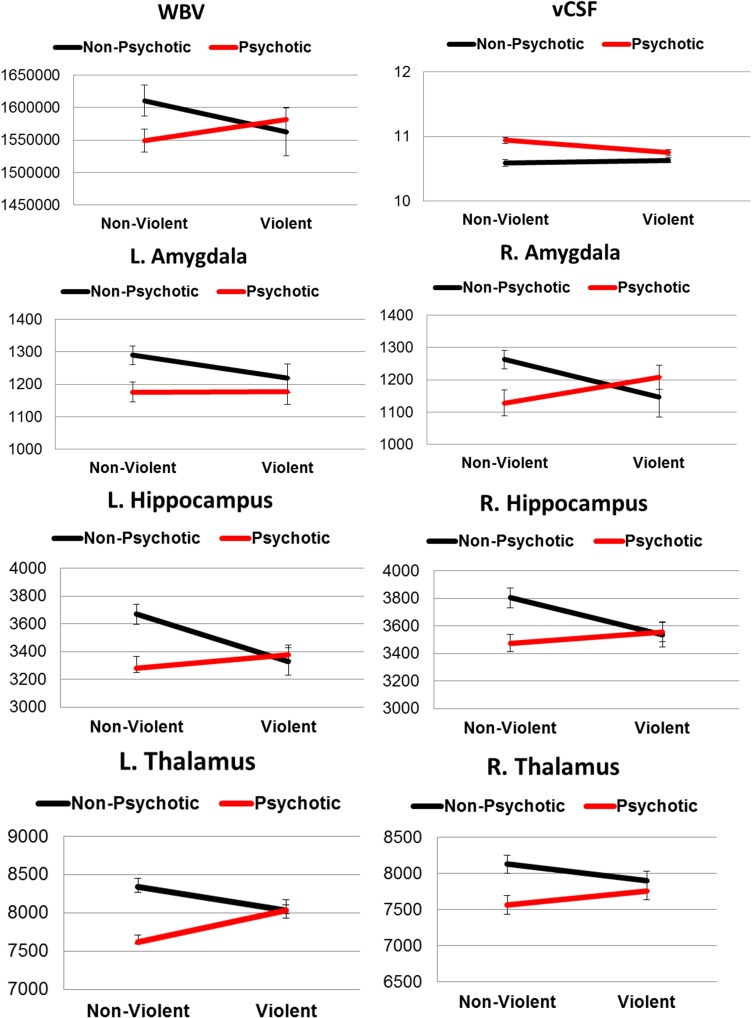
ROI mean volumes for each group. Vertical error bars indicate standard error of the mean.

Fig 2 illustrates the effects of violence and diagnosis on brain region volumes.

#### Left and Right Amygdala

The ANOVA for left amygdala indicated a trend towards a main effect for diagnosis (F_1,109_ = 3.89, p = .051). For the right amygdala, a significant interaction between diagnosis and violence was found (F_1,109_ = 5.3, p = .023). However, only a trend towards an interaction was observed after including substance abuse as a covariate (*F*_*1*,*109*_ = 3.679, *p* = .05). The interaction appeared to be driven by a double dissociation between factors, with decreased volume in NPV relative to HC and increased volume in VS relative to NVS. Bonferroni corrected post hoc pairwise comparison using independent samples t-test revealed no differences between groups.

Table 4 lists results obtained by analysis of variance.

**Table 4:** ANOVA; f/p values are listed for main and interactive effects for each ROI.

ROI	DF	Diagnosis	Violence	Diagnosis x Violence
WBV	1	1.492 (.*225*)	.829 (.*365*)	2.39 (.*125*)
vCSF	1	27.89 (*<* .0001***)	.140 (.*709*)	5.82 (.018*)
L. Amygdala	1	3.89 (.*051*)	.045 (.*832*)	.554 (.*459*)
R. Amygdala	1	.402 (.*527*)	.505 (.*479*)	3.679 (.*058*)
L. Hippocampus	1	4.09 (.046*)	2.01 (.*160*)	5.46 (.021*)
R. Hippocampus	1	3.51 (.*064*)	.609 (.*437*)	3.73 (.*056*)
L. Thalamus	1	11.12 (.001**)	.211 (.*647*)	8.36 (.005*)
R. Thalamus	1	7.69 (.007*)	.724 (.397)	1.45 (.232)
Error	109			

ANOVAs using WRAT-4 T-scores, Age, and SUD as covariates.

* *P* ≤ 0.05

** *P* ≤ 0.001

*** *P* ≤.0001. Columns includes degree of freedom (DF), F-scores and *p*-values (italicized).

#### Left and Right Hippocampus

For the left hippocampus volume, there was a main effect of diagnosis (*F*_1,109_ = 4.08, *p* = 0.04) with reduction seen for patients with schizophrenia. There was also an interaction between diagnosis and violence (*F*_1,109_ = 5.46, *p* = 0.02). Corrected post hoc pairwise comparisons revealed a group difference between NVS and HC (*p* = .026) for left hippocampal volume. No effects were found for the right hippocampus.

#### Left and Right Thalamus

For both left and right thalamus, ANOVA revealed a main effect for diagnosis (LT: *F*_1,109_ = 11.12, *p* = 0.001; RT: *F*_1,109_ = 7.69, *p* = 0.007) with reduction seen in patients with schizophrenia. Furthermore, a significant interaction between diagnosis and violence was observed for the left thalamus (*F*_1,109_ = 8.35, *p* = 0.005). Post hoc pairwise comparisons revealed a group difference between NVS and HC (*p* < .001).

#### Ventricular Cerebrospinal Fluid

Due to issues of skewness, vCSF was transformed using a natural log to approximate a Gaussian distribution. Analysis indicated a main effect for diagnosis (F_1,109_ = 27.88, p = 0.001) with increased vCSF volume for patients with schizophrenia. Furthermore, there was a significant interaction between diagnosis and violence (F_1,109_ = 5.82, p = 0.01). Post hoc pairwise comparisons revealed increased volume in NVS (p < .001) and VS (p = .008) relative to HC, as well as increased volume in NVS relative to NPV (*p* = .002).

#### The Role of Negative Symptoms and Medication

Here the analysis was limited to patients with a diagnosis of schizophrenia to investigate the potential role of several factors, such as medication, negative symptoms, and illness duration on gray matter volume loss. As indicated in Table 2, there was no group difference for CPZ equivalent. Further, CPZ equivalent did not correlate with WBV (*p* = .79), vCSF (*p* = .26), left amygdala (*p* = .43), left hippocampus (*p* = .71), left thalamus (*p* = .84), right amygdala (*p* = .93), right hippocampus (*p* = .94), or right thalamus (*p* = .87) volumes. CPZ equivalent values were therefore not added as a covariate. Groups did differ on age, academic achievement, substance use, as well as negative symptoms, and these variables were therefore included as covariates in our analysis. For this subsample analysis including only patients with schizophrenia, significant differences between patients with and without a history of violence were only found for vCSF with increased volume seen for NVS (*F*_1,59_ = 4.63, *p* = 0.03).

## Discussion

We set out to investigate structural characteristics in schizophrenia patients with and without a history of violence, and compare them to non-psychotic violent and healthy control individuals. Our goals were two-fold, to probe for volumetric abnormalities linked to aggression and to determine possible differences of such abnormalities between aggressive individuals with and without psychotic disorder. One particular interest pertained to amygdala volume as there are somewhat conflicting results implicating this region in violence. Out of four studies [24, 26, 30, 31], only one reported an association between amygdala reduction and violence in patients with schizophrenia. At the same time, decreased amygdala volume has been linked to a variety of mental disorders characterized by high aggression, including conduct disorder [59]; for a recent meta-analysis see [60] and psychopathy [61, 62].

In our study, we found several regions with structural abnormalities in patients with schizophrenia. Patients showed elevated vCSF volume and smaller left hippocampus as well as left thalamus volumes, particularly in individuals without a history of violence [63, 64]. Furthermore, patients had reduction in right thalamus size and a strong trend towards smaller left amygdala volume was found. With regard to right amygdala, our findings indicated an interaction between violence and diagnosis. The effect appeared to be driven by a double dissociation. That is, reductions associated with violence were seen for non-psychotic individuals, while for psychotic patient’s reductions were observed in non-violent individuals. Importantly, after accounting for substance abuse in our analysis only a trend towards an interaction was observed.

Overall, our findings in patients are in line with a recent large-scale study of subcortical brain regions in 2028 individuals with schizophrenia [65]. Among other structures, abnormalities were found for hippocampus, amygdala, thalamus, and lateral ventricle volumes. Evidence by us and others underscore widespread morphometric abnormalities in subcortical regions possible related to aberrant integration and information transfer processes in schizophrenia. Also, recent efforts to establish temporal lobe abnormalities as confirmatory biomarkers among youth at high-risk for development of psychosis have produced promising results [66]. One open question concerns stronger abnormalities in non-violent patients relative to their violent peers in our data.

With regard to our substance abuse finding it is important to note that a recent meta-analysis of studies probing violence in individuals on the psychosis spectrum concluded that most of the elevated risk was driven by patients with substance abuse comorbidity [67]. Here we report, for the first time according to our knowledge, that substance abuse might play a mediating role in the association between amygdala reduction and violence in non-psychotic violent individuals.

Considering all aggressive individuals in both the VS and NPV group, we found no evidence for shared structural abnormalities underlying violence. However, there might be other morphological markers and/or other regions of interest that might be important to consider to determine common structural abnormalities linked to violence. Furthermore, we found no evidence for amygdala volume reduction in violent patients with schizophrenia. Previous studies also reported no reductions in violent patients [24–26], but questions remained as to whether these studies were sufficiently powered to detect differences. Our sample was between two and three times larger than the ones in previous schizophrenia studies with a focus on violence [24–26], which provides a measure of confidence that amygdala size might not be part of the structural abnormalities commonly associated with violence, at least in schizophrenia.

A limitation of the current study is the use of a 1.5T scanner, which does not provide the same level of spatial resolution as more modern 3T scanners. Our sample is comprised of chronic patients with schizophrenia and therefore generalizations to patients in their early stages of illness might not be appropriate. Additionally, the self-report nature of the Buss-Perry, which has been found to be influenced by factors of social desirability [43] could possibly lead to lower scores, although the LHA scale is not based on self-report and is immune from concerns of social desirability. Utilizing the LHA scale for grouping purposes is a strength of the current report because of this consideration. Another strength of the study is the use of an automated approach to segmentation (FSL's FIRST), which has been reported to be a reliable and valid method of subcortical segmentation [68], and is widely used by researchers in various clinical populations [37, 69, 70]. A final, and important strength, is the relatively large sample of clinical patients and control participants used in this study, with the former demonstrating aberrant processing of emotionally salient images [29].

Future research should focus on other areas of grey matter that could be related to the elevated rates of violence in patients with schizophrenia. In this study, due to select *a priori* regions of interest, and limited regions within the FIRST library, the following regions were not investigated. One such region is the superior temporal sulcus (STS), a region associated with facial recognition [71, 72] which has direct projections to the amygdala [73], and which has been found to be impaired in the schizophrenia population [74, 75]. Another region of interest is the striatum, which includes the putamen, previously found to be enlarged in the VS group [24]. The putamen is part of the FIRST library but was not included in this analysis because of *a priori* ROI selection. OFC and PFC regions have been previously implicated in aggression and schizophrenia and should, in concert with subcortical regions, be considered in future research. Additionally, large scale neuroimaging and electrophysiology studies involving genetics (e.g. MAO-A and COM-T) and their role in brain structure and function could be helpful in delineating differences between the VS and NVS groups from NPV individuals. Both MAO-A and COM-T genes have been previously associated with violence and aggression [76–81], and both have been linked with schizophrenia [82–89].

In summary, our ROI analysis of temporal and subcortical regions confirmed widespread morphometric brain abnormalities in patients with schizophrenia. We found no evidence for smaller amygdala size in violent patients with schizophrenia. Finally, smaller amygdala size in violent non-psychotic individuals was mostly accounted by substance abuse problems within this group. The latter results might provide indication that substance abuse is a potential mediator in the relationship between brain abnormalities and aggression in non-psychotic individuals. Overall, our results underscore the importance of volumetric abnormalities in aggression and provide some indication for distinct brain regions in the characterization of psychotic and non-psychotic violent individuals.

## Supporting Information

S1 Table Anonymized data. (XLSX)

## References

[1] Swanson JW, Swartz MS, Van Dorn RA, Elbogen EB, Wagner HR, Rosenheck RA, et al A national study of violent behavior in persons with schizophrenia. Archives of general psychiatry. 2006;63(5):490–9. 10.1001/archpsyc.63.5.490 16651506

[2] Mullen PE, Burgess P, Wallace C, Palmer S, Ruschena D. Community care and criminal offending in schizophrenia. The Lancet. 2000;355(9204):614–7. 10.1016/S0140-6736(99)05082-5 10696982

[3] Nederlof AF, Muris P, Hovens JE. The epidemiology of violent behavior in patients with a psychotic disorder: A systematic review of studies since 1980. Aggression and violent behavior. 2013;18(1):183–9.

[4] Krakowski MI, Czobor P. Depression and impulsivity as pathways to violence: implications for antiaggressive treatment. Schizophrenia bulletin. 2014;40(4):886–94. PubMed Central PMCID: PMC4059442. 10.1093/schbul/sbt117 23943412 PMC4059442

[5] Davidson RJ, Putnam KM, Larson CL. Dysfunction in the neural circuitry of emotion regulation—a possible prelude to violence. Science. 2000;289(5479):591–4. Epub 2000/08/01. 10915615 10.1126/science.289.5479.591

[6] Terri Roberton MD, Romola S. Bucks. Emotion regulation and aggression. Aggression and Violent Behavior. 2012;17(1):72–82.

[7] Van Elst LT, Woermann F, Lemieux L, Thompson P, Trimble M. Affective aggression in patients with temporal lobe epilepsy. Brain: a journal of neurology. 2000;123(2):234–43. 10648432 10.1093/brain/123.2.234

[8] Bufkin JL, Luttrell VR. Neuroimaging studies of aggressive and violent behavior current findings and implications for criminology and criminal justice. Trauma, Violence, & Abuse. 2005;6(2):176–91. 10.1177/1524838005275089 15753199

[9] Coccaro EF. Neurotransmitter correlates of impulsive aggression in humans. Annals of the New York Academy of Sciences. 1996;794(1):82–9. 8853594 10.1111/j.1749-6632.1996.tb32511.x

[10] Krakowski M. Violence and serotonin: influence of impulse control, affect regulation, and social functioning. The Journal of neuropsychiatry and clinical neurosciences. 2003. 10.1176/jnp.15.3.294 12928505

[11] Coccaro EF, Fanning JR, Phan KL, Lee R. Serotonin and impulsive aggression. CNS spectrums. 2015;20(03):295–302. 25997605 10.1017/S1092852915000310

[12] Blair RJ. Applying a cognitive neuroscience perspective to the disorder of psychopathy. Development and psychopathology. 2005;17(3):865–91. 10.1017/S0954579405050418 16262996

[13] Kiehl KA. A cognitive neuroscience perspective on psychopathy: evidence for paralimbic system dysfunction. Psychiatry research. 2006;142(2–3):107–28. PubMed Central PMCID: PMC2765815. 10.1016/j.psychres.2005.09.013 16712954 PMC2765815

[14] Siever LJ. Neurobiology of aggression and violence. The American journal of psychiatry. 2008;165(4):429–42. PubMed Central PMCID: PMC4176893. 10.1176/appi.ajp.2008.07111774 18346997 PMC4176893

[15] DeWall CN, Finkel EJ, Denson TF. Self‐control inhibits aggression. Social and Personality Psychology Compass. 2011;5(7):458–72.

[16] Denson TF, DeWall CN, Finkel EJ. Self-control and aggression. Current Directions in Psychological Science. 2012;21(1):20–5.

[17] Hoptman MJ, Antonius D, Mauro CJ, Parker EM, Javitt DC. Cortical thinning, functional connectivity, and mood-related impulsivity in schizophrenia: relationship to aggressive attitudes and behavior. The American journal of psychiatry. 2014;171(9):939–48. PubMed Central PMCID: PMC4178944. 10.1176/appi.ajp.2014.13111553 25073506 PMC4178944

[18] Heatherton TF. Neuroscience of self and self-regulation. Annual review of psychology. 2011;62:363 10.1146/annurev.psych.121208.131616 21126181 PMC3056504

[19] Niu L, Matsui M, Zhou S-Y, Hagino H, Takahashi T, Yoneyama E, et al Volume reduction of the amygdala in patients with schizophrenia: a magnetic resonance imaging study. Psychiatry Research: Neuroimaging. 2004;132(1):41–51. 10.1016/j.pscychresns.2004.06.002 15546702

[20] Steen RG, Mull C, Mcclure R, Hamer RM, Lieberman JA. Brain volume in first-episode schizophrenia Systematic review and meta-analysis of magnetic resonance imaging studies. The British Journal of Psychiatry. 2006;188(6):510–8. 16738340 10.1192/bjp.188.6.510

[21] Velakoulis D, Wood SJ, Wong MT, McGorry PD, Yung A, Phillips L, et al Hippocampal and amygdala volumes according to psychosis stage and diagnosis: A magnetic resonance imaging study of chronic schizophrenia, first-episode psychosis, and ultra–high-risk individuals. Archives of general psychiatry. 2006;63(2):139–49. 10.1001/archpsyc.63.2.139 16461856

[22] Bora E, Fornito A, Radua J, Walterfang M, Seal M, Wood SJ, et al Neuroanatomical abnormalities in schizophrenia: a multimodal voxelwise meta-analysis and meta-regression analysis. Schizophrenia research. 2011;127(1):46–57. 21300524 10.1016/j.schres.2010.12.020

[23] Shepherd AM, Laurens KR, Matheson SL, Carr VJ, Green MJ. Systematic meta-review and quality assessment of the structural brain alterations in schizophrenia. Neuroscience & Biobehavioral Reviews. 2012;36(4):1342–56. 22244985 10.1016/j.neubiorev.2011.12.015

[24] Barkataki I, Kumari V, Das M, Taylor P, Sharma T. Volumetric structural brain abnormalities in men with schizophrenia or antisocial personality disorder. Behavioural brain research. 2006;169(2):239–47. Epub 2006/02/10. 10.1016/j.bbr.2006.01.009 16466814

[25] Kumari V, Barkataki I, Goswami S, Flora S, Das M, Taylor P. Dysfunctional, but not functional, impulsivity is associated with a history of seriously violent behaviour and reduced orbitofrontal and hippocampal volumes in schizophrenia. Psychiatry Research: Neuroimaging. 2009;173(1):39–44. 10.1016/j.pscychresns.2008.09.003 19442493

[26] Kumari V, Gudjonsson GH, Raghuvanshi S, Barkataki I, Taylor P, Sumich A, et al Reduced thalamic volume in men with antisocial personality disorder or schizophrenia and a history of serious violence and childhood abuse. European psychiatry: the journal of the Association of European Psychiatrists. 2013;28(4):225–34. Epub 2012/09/05. 22944337 10.1016/j.eurpsy.2012.03.002

[27] Yang Y, Raine A, Han CB, Schug RA, Toga AW, Narr KL. Reduced hippocampal and parahippocampal volumes in murderers with schizophrenia. Psychiatry research. 2010;182(1):9–13. PubMed Central PMCID: PMC2855857. 10.1016/j.pscychresns.2009.10.013 20227253 PMC2855857

[28] Hoptman MJ, D'Angelo D, Catalano D, Mauro CJ, Shehzad ZE, Kelly AM, et al Amygdalofrontal functional disconnectivity and aggression in schizophrenia. Schizophrenia bulletin. 2010;36(5):1020–8. Epub 2009/04/02. PubMed Central PMCID: PMC2930349. 10.1093/schbul/sbp012 19336392 PMC2930349

[29] De Sanctis P, Foxe JJ, Czobor P, Wylie GR, Kamiel SM, Huening J, et al Early sensory-perceptual processing deficits for affectively valenced inputs are more pronounced in schizophrenia patients with a history of violence than in their non-violent peers. Social cognitive and affective neuroscience. 2012. Epub 2012/05/09. 10.1093/scan/nss052 PMC3739916 22563006

[30] Wong M, Lumsden J, Fenton G, Fenwick P. Electroencephalography, computed tomography and violence ratings of male patients in a maximum‐security mental hospital. Acta Psychiatrica Scandinavica. 1994;90(2):97–101. 7976465 10.1111/j.1600-0447.1994.tb01562.x

[31] Kumari V, Das M, Taylor PJ, Barkataki I, Andrew C, Sumich A, et al Neural and behavioural responses to threat in men with a history of serious violence and schizophrenia or antisocial personality disorder. Schizophrenia research. 2009;110(1):47–58. 19230621 10.1016/j.schres.2009.01.009

[32] Derogatis L, Lipman R. SCL-90. Administration, scoring and procedures manual-I for the R (revised) version and other instruments of the Psychopathology Rating Scales Series Chicago: Johns Hopkins University School of Medicine 1977.

[33] Matthies S, Rusch N, Weber M, Lieb K, Philipsen A, Tuescher O, et al Small amygdala-high aggression? The role of the amygdala in modulating aggression in healthy subjects. The world journal of biological psychiatry: the official journal of the World Federation of Societies of Biological Psychiatry. 2012;13(1):75–81. 10.3109/15622975.2010.541282 22256828

[34] Bobes Leon MA, Ostroski F, Diaz K, Romero C, Borja K, Santos Y, et al, editors. Functional and structural impairment of the left amygdala in violent non-psychopath men. Psychophysiology; 2013.

[35] Raine A, Dodge K, Loeber R, Gatzke-Kopp L, Lynam D, Reynolds C, et al The Reactive-Proactive Aggression Questionnaire: Differential Correlates of Reactive and Proactive Aggression in Adolescent Boys. Aggressive behavior. 2006;32(2):159–71. PubMed Central PMCID: PMC2927832. 10.1002/ab.20115 20798781 PMC2927832

[36] Salekin RT, Rogers R, Sewell KW. A review and meta‐analysis of the psychopathy checklist and psychopathy checklist‐revised: predictive validity of dangerousness. Clinical Psychology: Science and Practice. 1996;3(3):203–15.

[37] Pardini DA, Raine A, Erickson K, Loeber R. Lower amygdala volume in men is associated with childhood aggression, early psychopathic traits, and future violence. Biological psychiatry. 2014;75(1):73–80. PubMed Central PMCID: PMC3751993. 10.1016/j.biopsych.2013.04.003 23647988 PMC3751993

[38] Krakowski MI, De Sanctis P, Foxe JJ, Hoptman MJ, Nolan K, Kamiel S, et al Disturbances in Response Inhibition and Emotional Processing as Potential Pathways to Violence in Schizophrenia: A High-Density Event-Related Potential Study. Schizophrenia bulletin. 2016;42(4):963–74. PubMed Central PMCID: PMCPMC4903062. 10.1093/schbul/sbw005 26895845 PMC4903062

[39] First MB, Spitzer RL, Gibbon M, Williams JB. User's guide for the Structured clinical interview for DSM-IV axis I disorders SCID-I: clinician version: American Psychiatric Pub; 1997.

[40] Association AP. Diagnostic and statistical manual-text revision (DSM-IV-TRim, 2000): American Psychiatric Association; 2000.

[41] Coccaro EF, Berman ME, Kavoussi RJ. Assessment of life history of aggression: development and psychometric characteristics. Psychiatry research. 1997;73(3):147–57. 9481806 10.1016/s0165-1781(97)00119-4

[42] Buss AH, Perry M. The aggression questionnaire. Journal of personality and social psychology. 1992;63(3):452–9. 1403624 10.1037//0022-3514.63.3.452

[43] Harris JA. A further evaluation of the Aggression Questionnaire: Issues of validity and reliability. Behaviour research and therapy. 1997;35(11):1047–53. 9431736 10.1016/s0005-7967(97)00064-8

[44] Patton JH, Stanford MS. Factor structure of the Barratt impulsiveness scale. Journal of clinical psychology. 1995;51(6):768–74. 8778124 10.1002/1097-4679(199511)51:6<768::aid-jclp2270510607>3.0.co;2-1

[45] Stanford MS, Mathias CW, Dougherty DM, Lake SL, Anderson NE, Patton JH. Fifty years of the Barratt Impulsiveness Scale: An update and review. Personality and individual differences. 2009;47(5):385–95.

[46] Wilkinson GS, Robertson GJ. WRAT 4: Wide Range Achievement Test; professional manual: Psychological Assessment Resources, Incorporated; 2006.

[47] Kay SR, Flszbein A, Opfer LA. The positive and negative syndrome scale (PANSS) for schizophrenia. Schizophrenia bulletin. 1987;13(2):261 3616518 10.1093/schbul/13.2.261

[48] Peralta V, Cuesta MJ. Psychometric properties of the positive and negative syndrome scale (PANSS) in schizophrenia. Psychiatry research. 1994;53(1):31–40. 7991730 10.1016/0165-1781(94)90093-0

[49] Morey RA, Petty CM, Xu Y, Hayes JP, Wagner HR 2nd, Lewis DV, et al A comparison of automated segmentation and manual tracing for quantifying hippocampal and amygdala volumes. NeuroImage. 2009;45(3):855–66. PubMed Central PMCID: PMCPMC2714773. 10.1016/j.neuroimage.2008.12.033 19162198 PMC2714773

[50] Patenaude B, Smith SM, Kennedy DN, Jenkinson M. A Bayesian model of shape and appearance for subcortical brain segmentation. NeuroImage. 2011;56(3):907–22. 10.1016/j.neuroimage.2011.02.046 21352927 PMC3417233

[51] Smith SM, De Stefano N, Jenkinson M, Matthews PM. Normalized accurate measurement of longitudinal brain change. Journal of computer assisted tomography. 2001;25(3):466–75. 11351200 10.1097/00004728-200105000-00022

[52] Smith SM, Jenkinson M, Woolrich MW, Beckmann CF, Behrens TE, Johansen-Berg H, et al Advances in functional and structural MR image analysis and implementation as FSL. NeuroImage. 2004;23:S208–S19. 10.1016/j.neuroimage.2004.07.051 15501092

[53] Jenkinson M, Smith S. A global optimisation method for robust affine registration of brain images. Medical image analysis. 2001;5(2):143–56. 11516708 10.1016/s1361-8415(01)00036-6

[54] Jenkinson M, Bannister P, Brady M, Smith S. Improved optimization for the robust and accurate linear registration and motion correction of brain images. NeuroImage. 2002;17(2):825–41. 12377157 10.1016/s1053-8119(02)91132-8

[55] Smith SM, Rao A, De Stefano N, Jenkinson M, Schott JM, Matthews PM, et al Longitudinal and cross-sectional analysis of atrophy in Alzheimer's disease: cross-validation of BSI, SIENA and SIENAX. NeuroImage. 2007;36(4):1200–6. 10.1016/j.neuroimage.2007.04.035 17537648

[56] Smith SM, Zhang Y, Jenkinson M, Chen J, Matthews P, Federico A, et al Accurate, robust, and automated longitudinal and cross-sectional brain change analysis. NeuroImage. 2002;17(1):479–89. 12482100 10.1006/nimg.2002.1040

[57] Smith SM, Rao A, De Stefano N, Jenkinson M, Schott JM, Matthews PM, et al Longitudinal and cross-sectional analysis of atrophy in Alzheimer's disease: cross-validation of BSI, SIENA and SIENAX. NeuroImage. 2007;36(4):1200–6. 10.1016/j.neuroimage.2007.04.035 17537648

[58] Kane JM, Leucht S, Carpenter D, Docherty JP, Expert Consensus Panel for Optimizing Pharmacologic Treatment of Psychotic D. The expert consensus guideline series. Optimizing pharmacologic treatment of psychotic disorders. Introduction: methods, commentary, and summary. The Journal of clinical psychiatry. 2003;64 Suppl 12:5–19. 14640142

[59] Fairchild G, Passamonti L, Hurford G, Hagan CC, von dem Hagen EA, van Goozen SH, et al Brain structure abnormalities in early-onset and adolescent-onset conduct disorder. The American journal of psychiatry. 2011;168(6):624–33. 10.1176/appi.ajp.2010.10081184 21454920

[60] Rogers JC, De Brito SA. Cortical and Subcortical Gray Matter Volume in Youths With Conduct Problems: A Meta-analysis. JAMA Psychiatry. 2016;73(1):64–72. 10.1001/jamapsychiatry.2015.2423 26650724

[61] Yang Y, Raine A, Narr KL, Colletti P, Toga AW. Localization of deformations within the amygdala in individuals with psychopathy. Archives of general psychiatry. 2009;66(9):986–94. PubMed Central PMCID: PMC3192811. 10.1001/archgenpsychiatry.2009.110 19736355 PMC3192811

[62] Ermer E, Cope LM, Nyalakanti PK, Calhoun VD, Kiehl KA. Aberrant paralimbic gray matter in criminal psychopathy. Journal of abnormal psychology. 2012;121(3):649–58. PubMed Central PMCID: PMC4039408. 10.1037/a0026371 22149911 PMC4039408

[63] Davatzikos C, Shen D, Gur RC, Wu X, Liu D, Fan Y, et al Whole-brain morphometric study of schizophrenia revealing a spatially complex set of focal abnormalities. Archives of general psychiatry. 2005;62(11):1218–27. 10.1001/archpsyc.62.11.1218 16275809

[64] Tanskanen P, Haapea M, Veijola J, Miettunen J, Jarvelin MR, Pyhtinen J, et al Volumes of brain, grey and white matter and cerebrospinal fluid in schizophrenia in the Northern Finland 1966 Birth Cohort: an epidemiological approach to analysis. Psychiatry research. 2009;174(2):116–20. 10.1016/j.pscychresns.2009.04.009 19853416

[65] van Erp TG, Hibar DP, Rasmussen JM, Glahn DC, Pearlson GD, Andreassen OA, et al Subcortical brain volume abnormalities in 2028 individuals with schizophrenia and 2540 healthy controls via the ENIGMA consortium. Molecular psychiatry. 2016;21(4):547–53. PubMed Central PMCID: PMCPMC4668237. 10.1038/mp.2015.63 26033243 PMC4668237

[66] Roalf DR, Quarmley M, Calkins ME, Satterthwaite TD, Ruparel K, Elliott MA, et al Temporal Lobe Volume Decrements in Psychosis Spectrum Youths. Schizophrenia bulletin. 2016. 10.1093/schbul/sbw112 PMC5463880 27559077

[67] Fazel S, Gulati G, Linsell L, Geddes JR, Grann M. Schizophrenia and violence: systematic review and meta-analysis. PLoS Med. 2009;6(8):e1000120 PubMed Central PMCID: PMCPMC2718581. 10.1371/journal.pmed.1000120 19668362 PMC2718581

[68] Morey RA, Selgrade ES, Wagner HR, Huettel SA, Wang L, McCarthy G. Scan–rescan reliability of subcortical brain volumes derived from automated segmentation. Human brain mapping. 2010;31(11):1751–62. 10.1002/hbm.20973 20162602 PMC3782252

[69] De Jong L, Van der Hiele K, Veer I, Houwing J, Westendorp R, Bollen E, et al Strongly reduced volumes of putamen and thalamus in Alzheimer's disease: an MRI study. Brain: a journal of neurology. 2008;131(12):3277–85. 19022861 10.1093/brain/awn278 PMC2639208

[70] Sexton CE, Mackay CE, Lonie JA, Bastin ME, Terrière E, O'Carroll RE, et al MRI correlates of episodic memory in Alzheimer's disease, mild cognitive impairment, and healthy aging. Psychiatry Research: Neuroimaging. 2010;184(1):57–62. 10.1016/j.pscychresns.2010.07.005 20832251

[71] Baylis G, Rolls ET, Leonard C. Selectivity between faces in the responses of a population of neurons in the cortex in the superior temporal sulcus of the monkey. Brain research. 1985;342(1):91–102. 4041820 10.1016/0006-8993(85)91356-3

[72] Iidaka T. Role of the fusiform gyrus and superior temporal sulcus in face perception and recognition: An empirical review. Japanese Psychological Research. 2014;56(1):33–45.

[73] Aggleton J, Burton M, Passingham R. Cortical and subcortical afferents to the amygdala of the rhesus monkey (Macaca mulatta). Brain research. 1980;190(2):347–68. 6768425 10.1016/0006-8993(80)90279-6

[74] Rajarethinam R, DeQuardo J, Nalepa R, Tandon R. Superior temporal gyrus in schizophrenia: a volumetric magnetic resonance imaging study. Schizophrenia research. 2000;41(2):303–12. 10708339 10.1016/s0920-9964(99)00083-3

[75] Kim J-J, Crespo-Facorro B, Andreasen NC, O'Leary DS, Magnotta V, Nopoulos P. Morphology of the lateral superior temporal gyrus in neuroleptic naıve patients with schizophrenia: relationship to symptoms. Schizophrenia research. 2003;60(2):173–81. 12591581 10.1016/s0920-9964(02)00299-2

[76] Hygen BW, Belsky J, Stenseng F, Lydersen S, Guzey IC, Wichstrøm L. Child exposure to serious life events, COMT, and aggression: Testing differential susceptibility theory. Developmental psychology. 2015;51(8):1098 10.1037/dev0000020 26053146

[77] Veroude K, Zhang‐James Y, Fernàndez‐Castillo N, Bakker MJ, Cormand B, Faraone SV. Genetics of aggressive behavior: An overview. American Journal of Medical Genetics Part B: Neuropsychiatric Genetics. 2015. 10.1002/ajmg.b.32364 26345359

[78] Kotler M, Barak P, Cohen H, Averbuch IE, Grinshpoon A, Gritsenko I, et al Homicidal behavior in schizophrenia associated with a genetic polymorphism determining low catechol O‐methyltransferase (COMT) activity. American journal of medical genetics. 1999;88(6):628–33. 10581481

[79] Widom CS, Brzustowicz LM. MAOA and the “cycle of violence:” childhood abuse and neglect, MAOA genotype, and risk for violent and antisocial behavior. Biological psychiatry. 2006;60(7):684–9. 10.1016/j.biopsych.2006.03.039 16814261

[80] Buckholtz JW, Meyer-Lindenberg A. MAOA and the neurogenetic architecture of human aggression. Trends in neurosciences. 2008;31(3):120–9. 10.1016/j.tins.2007.12.006 18258310

[81] Frazzetto G, Di Lorenzo G, Carola V, Proietti L, Sokolowska E, Siracusano A, et al Early trauma and increased risk for physical aggression during adulthood: the moderating role of MAOA genotype. PloS one. 2007;2(5):e486 10.1371/journal.pone.0000486 17534436 PMC1872046

[82] Shifman S, Bronstein M, Sternfeld M, Pisanté-Shalom A, Lev-Lehman E, Weizman A, et al A highly significant association between a COMT haplotype and schizophrenia. The American Journal of Human Genetics. 2002;71(6):1296–302. 10.1086/344514 12402217 PMC378567

[83] Shifman S, Bronstein M, Sternfeld M, Pisanté A, Weizman A, Reznik I, et al COMT: a common susceptibility gene in bipolar disorder and schizophrenia. American Journal of Medical Genetics Part B: Neuropsychiatric Genetics. 2004;128(1):61–4. 10.1002/ajmg.b.30032 15211633

[84] Li T, Sham P, Vallada H, Xie T, Tang X, Murray R, et al Preferential transmission of the high activity allele of COMT in schizophrenia. Psychiatric genetics. 1996;6(3):131–4. 8902889 10.1097/00041444-199623000-00005

[85] Bilder RM, Volavka J, ál Czobor P, Malhotra AK, Kennedy JL, Ni X, et al Neurocognitive correlates of the COMT Val 158 Met polymorphism in chronic schizophrenia. Biological psychiatry. 2002;52(7):701–7. 12372660 10.1016/s0006-3223(02)01416-6

[86] Zammit S, Jones G, Jones SJ, Norton N, Sanders RD, Milham C, et al Polymorphisms in the MAOA, MAOB, and COMT genes and aggressive behavior in schizophrenia. American Journal of Medical Genetics Part B: Neuropsychiatric Genetics. 2004;128(1):19–20. 10.1002/ajmg.b.30021 15211623

[87] De Luca V, Tharmalingam S, Müller DJ, Wong G, de Bartolomeis A, Kennedy JL. Gene–gene interaction between MAOA and COMT in suicidal behavior: Analysis in schizophrenia. Brain research. 2006;1097(1):26–30. 10.1016/j.brainres.2006.04.053 16725119

[88] Chen Y, Zhang J, Zhang L, Shen Y, Xu Q. Effects of MAOA promoter methylation on susceptibility to paranoid schizophrenia. Human genetics. 2012;131(7):1081–7. 10.1007/s00439-011-1131-5 22198720

[89] Jönsson EG, Norton N, Forslund K, Mattila-Evenden M, Rylander G, Åsberg M, et al Association between a promoter variant in the monoamine oxidase A gene and schizophrenia. Schizophrenia research. 2003;61(1):31–7. 12648733 10.1016/s0920-9964(02)00224-4

